# Head-to-head: meropenem/vaborbactam versus ceftazidime/avibactam in ICUs patients with KPC-producing *K. pneumoniae* infections– results from a retrospective multicentre study

**DOI:** 10.1007/s15010-025-02608-7

**Published:** 2025-07-16

**Authors:** Andrea Marino, Alberto Enrico Maraolo, Maria Mazzitelli, Alessandra Oliva, Nicholas Geremia, Andrea De Vito, Chiara Gullotta, Vincenzo Scaglione, Eleonora Vania, Sara Lo Menzo, Paolo Navalesi, Lorenzo Volpicelli, Andrea Fiori, Pamela Prestifilippo, Annamaria Cattelan, Claudio Maria Mastroianni, Giordano Madeddu, Bruno Cacopardo, Giuseppe Nunnari

**Affiliations:** 1 Department of Clinical and Experimental Medicine, Infectious Diseases Unit, ARNAS Garibaldi Hospital, University of Catania, Catania, Italy; 2 Section of Infectious Diseases, Department of Clinical Medicine and Surgery, University of Naples “Federico II”, Naples, Italy; 3 Infectious and Tropical Diseases Unit, Padua University Hospital, Padua, Italy; 4 Department of Public Health and Infectious Diseases, Sapienza University of Rome, Rome, Italy; 5 Unit of Infectious Diseases, Department of Clinical Medicine, Ospedale “dell’Angelo”, Venice, Italy; 6 Unit of Infectious Diseases, Department of Medicine, Surgery and Pharmacy, University of Sassari, Sassari, Italy; 7 Department of Medicine (DIMED), University of Padua, Padua, Italy; 8 Institute of Anesthesia and Intensive Care, Padua University Hospital, Padua, Italy; 9 School of Medicine, University of Sassari, Sassari, Italy; 10 Intensive Care Unit, ARNAS Garibaldi Hospital, Catania, Italy

**Keywords:** Enterobacetrales, Meropenem/vaborbactam, Ceftazidime/avibactam, Klebsiella pneumoniae, KPC, ICU patients

## Abstract

**Purpose:**

Meropenem/vaborbactam (M/V) and ceftazidime/avibactam (C/A) are considered key agents in treating KPC-producing *Klebsiella pneumoniae (Kp)* infections. We compared these two drugs in ICUs patients with BSI and/or pneumoniae due to KPC- Kp.

**Methods:**

This retrospective multicentre study analysed ICU patients with bloodstream infections (BSI) and/or pneumonia caused by KPC-Kp across five Italian ICUs from January 2021 to December 2023. Propensity-score matching (PSM) was applied to mitigate the impact of confounding factors. The primary outcome was 30-day all-cause mortality. Secondary outcomes included early clinical improvement at 72 h, computing the odds ratio (OR) as effect size, and infection-related events. Subgroup analyses were performed based on relevant prognostic factors.

**Results:**

The study included 177 patients, with 88 subjects paired after-matching (52 treated with C/A and 36 with M/V). As for primary outcome, after PSM, no statistically significant differences in 30-day mortality were observed between the two groups: in the Kaplan-Meier survival log-rank test was *p* = 0.38, and PSM-adjusted HR of M/V on mortality was 0.65 (95% CI 0.55–1.68). As for secondary outcomes, M/V significantly improved early clinical response post-PSM (OR: 2.19, 95% CI: 1.35–3.55).

**Conclusions:**

M/V showed no statistically significant difference in 30-day mortality compared to C/A but demonstrated significantly improved in early clinical response for patients with KPC-Kp. These findings were consistent between unmatched and matched patients. Further prospective studies are warranted to validate these observations.

**Supplementary Information:**

The online version contains supplementary material available at 10.1007/s15010-025-02608-7.

## Background

Carbapenem-resistant Enterobacterales (CRE), particularly KPC producing *Klebsiella pneumoniae* (KPC-Kp), represent a critical threat in intensive care units (ICUs) due to limited treatment options and associated high morbidity and mortality rates [1–3]. Within this context, meropenem/vaborbactam (M/V) and ceftazidime/avibactam (C/A) have emerged as pivotal agents in managing infections caused by KPC-Kp, with both demonstrating efficacy in combating these challenging pathogens [4].

However, recent studies indicate varying resistance patterns and clinical outcomes between these agents, which underscore the need for comparative effectiveness assessments, particularly in ICU settings where infection rates and risks are exacerbated by patient vulnerabilities [5].

M/V, a combination of a carbapenem with a novel β-lactamase inhibitor, has shown strong efficacy against KPC-producing isolates, especially in cases resistant to ceftazidime/avibactam, suggesting it may serve as a second-line treatment where resistance to other agents is detected [6, 7]. C/A, on the other hand, has been favoured in treating CRE due to its activity against both KPC- and OXA-producing strains; however, increasing resistance among KPC-Kp strains raises concerns regarding its sustained efficacy, particularly in ICU patients [8, 9].

Although in vitro efficacy was compared in some works, only few studies directly compared in vivo these two drugs, and fewer assessed their use in ICU settings [10, 11].

This retrospective multicentre study aims to compare the clinical outcomes and mortality rates among ICU patients treated with M/V versus C/A for KPC-Kp infections. By analysing real-world data, this study will provide valuable insights into optimal therapeutic strategies for this critical patient population, potentially guiding antibiotic stewardship efforts in managing CRE infections in ICUs.

## Methods

### Study design and setting

This was a multi-centre, retrospective, observational cohort study involving 5 ICUs (Catania, Sassari, Roma, Padova, and Venezia). The study period was between January 2021 and December 2023.

### Participants

Clinical teams at each participating site identified potentially eligible patients using institutional databases and medical records. Inclusion criteria encompassed consecutive patients aged 18 years or older who were diagnosed with bloodstream infection (BSI) or pneumonia by *Klebsiella pneumoniae* carbapenemase (KPC)-producing, undergoing targeted treatment with M/V or C/A. Dose selection was at the discretion of the responsible clinicians. Patients were followed for up to 30 days from the date of the index culture. Exclusion criteria included invasive infections different from BSI and pneumonia, patients receiving less than 48 h of drugs due to early death within 2 days, strains resistant to drugs under investigation.

### Clinical variables and definitions

Demographic, clinical, and microbiological data were collected from hospital medical records. Recorded data included age, body mass index (BMI) and sex; comorbidities and severity of underlying diseases assessed using the Charlson Comorbidity Index [12].

Severity of illness was calculated using the Sequential Organ Failure Assessment (SOFA) score [13] and APACHE II score [14].

KPC-Kp BSI was defined when KPC-Kp was isolated from BCs in the presence of clinical signs of infection and BSI onset was defined as the date of collection of the index BC.

Bloodstream infection was defined as the presence of at least one set of positive blood cultures (BC) and pneumonia was defined as presence of imaging suggestive of pneumonia along with symptoms, signs, and biochemical parameters suggestive for pneumonia [15].

Hospital acquired/ventilator-associated pneumonia (HAP/VAP) were defined in accordance with CDC/NHSN surveillance definition of healthcare-associated infection for pneumonia with specific criteria.

Combination therapy was defined as the use of two or more drugs for at least 48 h.

Early (within 48–72 h) clinical improvement was defined as at least one of the following: weaning from vasopressors; fever disappearance > 48 h; procalcitonin reduction by > 80%; and C-reactive protein reduction by > 75% [9, 16, 17].

The appropriateness of empirical treatment, defined as the antimicrobial therapy administered before susceptibility results were available, was evaluated when KPC-Kp was found to be susceptible to C/A and M/V, and when these drugs were administered within 48–72 h of the index blood culture collection.

### Microbiological studies

Bacterial isolates were identified following standard procedure. In line with the standard protocol of the hospitals’ Microbiology Laboratory aimed at expediting diagnostic procedures, bacterial pellets from positive blood cultures (BCs) were used for identification via MALDI-TOF MS (Bruker Daltonics). Molecular analysis to detect the blaKPC gene was subsequently conducted using the GeneXpert^®^ System (Cepheid). Antimicrobial susceptibility testing was carried out using either the VITEK 2 automated system (bioMérieux, Marcy l’Étoile, France) or the Sensititre™ system (Thermo Fisher Scientific).

### Outcomes

The primary outcome was all-cause mortality, assessed at 30-day, in the framework of a time-to-event analysis. The secondary outcome included early improvement at 72 h after therapy start. The incidence of *Clostridioides difficile* infection, infection relapses and candidemia were also evaluated as secondary outcomes but only descriptively.

### Statistical analysis

Descriptive statistics were generated, including means or medians with standard deviations for continuous variables, and frequencies and percentages for binary or categorical variables. The Wilcoxon rank-sum test was used to compare continuous variables, while Pearson’s chi-squared test or Fisher’s exact test when needed were applied to categorical variables. Differences were considered statistically significant if the *p*-value was less than 0.05.

#### Propensity score matching

To assess the effect of the use of one active agent opposed to another one, while ensuring an even distribution of important confounders between groups, we employed propensity-score matching [18]. The conduct and reporting of our propensity score model followed the recommendations provided by Eikenboom et al. [19].

Patients who received M/V or received C/A were matched 1:2 to reduce potential differences in characteristics deemed prognostic of clinical endpoints between the two groups.

The following covariates were included in the propensity-score model: age, sex, BMI as categorical variable (at least 30 or not), chronic obstructive pulmonary disease, oncohematology disease, diabetes mellitus, cardiovascular disease, cirrhosis, APACHE II score, nosocomial acquisition, mechanical ventilation, extracorporeal membrane oxygenation, dialysis, septic shock, infection type, polymicrobial infection.

Propensity-score–matched data were generated to estimate the average treatment effect among the overlap population (ATO), representing the effect of a treatment in a population at clinical equipoise, that is, eligible patients for whom either treatment is currently equally implemented or for whom there is no strong preference for one treatment over another [20]. Under the principle of equipoise, such patients are the ones most likely to be enrolled in a clinical trial [21]. The ATO addresses the question: “How would the outcomes differ, on average, if patients at clinical equipoise were given the treatment versus if the treatment were withheld?”

We explored different methods for generating propensity scores and used balance diagnostics [22] to assess the adequacy of matching, selecting the optimal method accordingly. Adequacy was evaluated by examining whether the distributions of measured baseline covariates were similar between the tested and untested groups, based on the standardized mean difference (SMD), with a threshold of 0.1 indicating acceptable covariate balance [23]. The values of absolute SMD of of covariates (both continuous and categorical) between groups were calculated by dividing the difference between the groups by their pooled standard deviation. Plot of standardized mean differences is presented in Figures S1 (available as supplementary data).

For the ATO estimand, we used a nearest-neighbour matching (1:2) without replacement and a caliper of 0.06 (the maximum allowed difference in propensity scores between matched subjects) [24], resorting to the covariate balancing propensity score (CBPS) algorithm, which is a type of logistic regression in which balance constraints are incorporated to a generalized method of moments estimation of the model coefficients [25].

#### Main analysis

Marginal effects, representing comparisons between the expected outcomes under treatment versus control conditions, were estimated and expressed as hazard ratios (HRs) and odds ratios (ORs) along with their 95% confidence intervals (CIs), as for the primary and the secondary outcome, respectively, both in the unmatched and in the matched sample, to determine the effectiveness of M/V versus C/A in the treatment of invasive infection by KPC-producing *K. pneumoniae*. Estimation was conducted via g-computation, incorporating a cluster-robust standard errors to adjust for paired observations, using the coefficient on treatment in a Cox model fit without covariates concerning the survival outcome, as well as fitting a model for the binary outcome given the treatment and the covariates used for computing the propensity score.

Patients lost to follow-up before the 30-day time frame owing to hospital discharge were right-censored in the time-to-event analysis.

Kaplan–Meier survival curves were plotted along with indication of log-rank test result to compare groups as far as time-to-event outcome were concerned.

All two-tailed *p* values less than 0.05 indicated statistical significance.

Analyses were performed in R, version 4.1.0 (R Core Team), using the packages *MatchIt*,* cobalt*,* survival*,* survminer* and *marginaleffects*.

#### Subgroup analyses

Marginal HRs were computed in the unmatched and in the matched sample across pre-defined subgroups in order to understand whether a treatment effect differs across levels of another variable: mechanical ventilation or not at baseline, presence or absence of septic shock, type of infection, the appropriateness of empirical treatment, the use of a combination therapy, modality of infusion of the drugs under investigation. Considering that there is no consensus about the best way to perform subgroup PSM in terms of balance, bias, or precision [26] two methods were applied: using the PSM cohort obtained for the main analysis in order to perform subgroup analysis, so retaining the same sample size but at risk of breaking the matched sets resulting in potential covariate imbalance (model A); carrying out matching in the full dataset but requiring exact matching on the moderator, thus creating a slightly different population (model B) [27]. Covariate balance plots related to model B for each treatment effect modifier are presented in the Supplementary material.

#### Sensitivity analyses for unmeasured confounding

E-values were calculated to assess the robustness of our findings regarding potential unmeasured confounders, representing the extent to which an unmeasured confounder would have to be associated with both treatment and outcome to invalidate the observed treatment-outcome association [28]. Greater E-values point at stronger treatment-outcome associations, as unmeasured confounders would have to have correspondingly large effect sizes to negate the results [21].

## Results

**Fig. 1:**
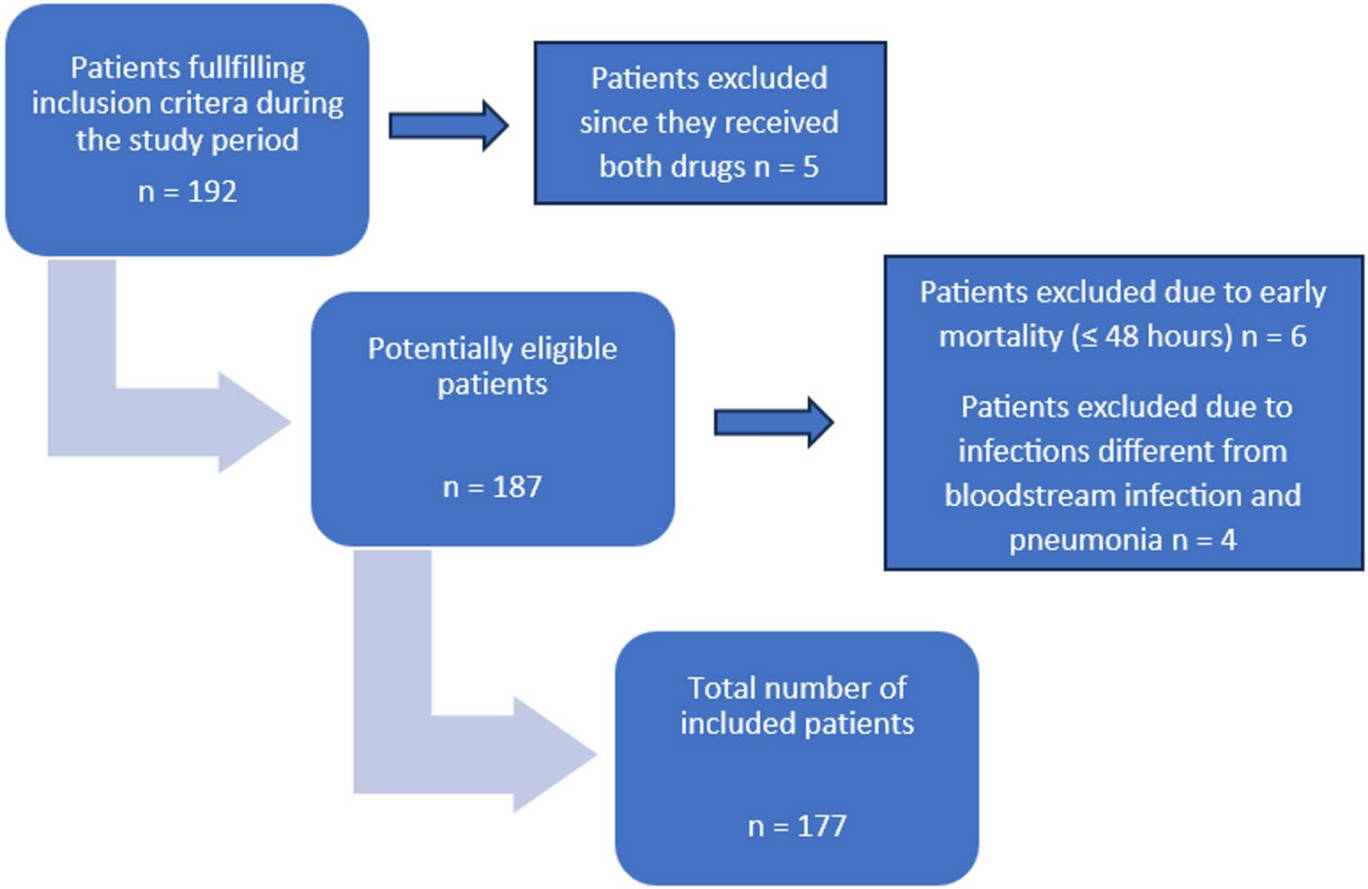
Flowchart of the patients included in the study

Of the 192 ICU patients identified with invasive infection by KPC-producing *Klebsiella pneumoniae*, treated with either M/V or C/A, 177 were included in the definitive analysis. Some patients were excluded due to overlap in treatment, defined as instances where patients received both drugs during their ICU stay, other were excluded due to infections in different sites from bloodstream and respiratory tract (Fig. 1).

### Baseline characteristics and propensity score matching

In the overall pre-matched cohort, 123 patients were treated with C/A, while 54 received M/V. The two groups were generally balanced in terms of factors such as age, sex, and body mass index (BMI). However, significant imbalances were observed in the pre-matched cohort for certain factors, including the presence of oncohematologic diseases (*p* = 0.04), chronic obstructive pulmonary disease (COPD) (*p* = 0.04), mechanical ventilation (*p* < 0.001), central venous catheter placement (*p* < 0.001), and shock (*p* < 0.001). Propensity score matching (PSM) was implemented to control for confounding variables, including patient demographics, clinical severity scores, and infection-related factors, resulting in comparable baseline characteristics between groups, as indicated by a standardized mean difference of < 0.1 for all covariates involved in the definition of the propensity score. After matching, the groups included 52 and 36 C/A and M/V patients, respectively. Table 1 showed the demographics and clinical characteristics of the two groups before and after PSM.

**Table 1:** – Demographics and clinical characteristics of patients treated with meropenem/vaborbactam and ceftazidime/avibactam in overall and propensity-matched cohorts

Variables	Pre-matched cohort				Post-matched cohort			
	C/A	M/V	p	smd	C/A	M/V	p	smd
	*N* = 123	*N* = 54			*N* = 52	*N* = 36		
Demographic information								
Age (median [IQR])	65 [55, 75]	63 [50, 70.75]	0.057	0.316	62 [49.75, 71]	61.00 [49.00, 70.25]	0.812	0.048
Gender Male = n (%)	59 (48.0)	27 (50.0)	0.932	0.041	29 (55.8)	19 (52.8)	0.953	0.060
Comorbidities								
BMI > 30 = n (%)	35 (28.5)	14 (25.9)	0.870	0.057	10 (19.2)	6 (16.7)	0.980	0.067
COPD = n (%)	19 (15.4)	16 (29.6)	0.048	0.344	8 (15.4)	5 (13.9)	1.000	0.042
CKD = n (%)	30 (24.4)	13 (24.1)	1.000	0.007	7 (13.5)	9 (25.0)	0.272	0.296
Oncohematology diseases = n (%)	15 (12.2)	14 (25.9)	0.040	0.355	9 (17.3)	8 (22.2)	0.765	0.124
DM = n (%)	35 (28.5)	12 (22.2)	0.497	0.144	11 (21.2)	6 (16.7)	0.803	0.115
Heart disease = n (%)	41 (33.3)	27 (50.0)	0.053	0.343	21 (40.4)	17 (47.2)	0.676	0.138
Cirrhosis = n (%)	6 (4.9)	1 (1.9)	0.594	0.168	2 (3.8)	1 (2.8)	1.000	0.060
Conditions at baseline								
APACHE II (median [IQR])	18.00 [12.00, 24.50]	17.00 [13.00, 23.00]	0.996	0.161	18.00 [13.50, 23.00]	17.00 [16.50, 17.00]	0.141	0.030
MV = n (%)	55 (44.7)	47 (87.0)	< 0.001	0.997	38 (73.1)	30 (83.3)	0.384	0.250
ECMO = n (%)	4 (3.3)	5 (9.3)	0.192	0.250	2 (3.8)	2 (5.6)	1.000	0.081
TPN = n (%)	67 (54.5)	37 (68.5)	0.114	0.292	30 (57.7)	24 (66.7)	0.530	0.186
Dialysis = n (%)	22 (17.9)	13 (24.5)	0.420	0.163	10 (19.2)	9 (25.0)	0.702	0.139
Deep vascular access = n (%)	123 (100.0)	53 (98.1)	0.671	0.194	52 (100.0)	35 (97.2)	0.852	0.239
Arterial line = n (%)	59 (89.4)	27 (100.0)	0.185	0.487	35 (89.7)	20 (100.0)	0.349	0.478
CVC = n (%)	88 (71.5)	54 (100.0)	< 0.001	0.892	44 (84.6)	36 (100.0)	0.037	0.603
PICC or midline = n (%)	33 (26.8)	14 (25.9)	1.000	0.020	12 (23.1)	11 (30.6)	0.590	0.169
PORT-A-CATH = n (%)	4 (3.3)	0 (0.0)	0.429	0.259	2 (3.8)	0 (0.0)	0.643	0.283
KPC-rectal-colonization = n (%)	112 (91.1)	52 (96.3)	0.359	0.217	47 (90.4)	34 (94.4)	0.771	0.154
COVID-19 = n (%)	13 (10.6)	4 (7.4)	0.704	0.111	4 (7.7)	3 (8.3)	1.000	0.024
sepsis = n (%)	114 (92.7)	48 (88.9)	0.588	0.131	47 (90.4)	31 (86.1)	0.780	0.133
shock = n (%)	46 (37.4)	39 (72.2)	< 0.001	0.747	31 (59.6)	23 (63.9)	0.855	0.088
Hospital acquired	112 (91.1)	50 (92.6)	0.964	0.056	47 (90.4)	33 (91.7)	1.000	0.045
Infection type			< 0.001	0.948			0.143	0.444
Pneumonia	9 (7.3)	12 (22.2)			6 (11.5)	7 (19.4)		
BSI plus pneumonia	79 (64.2)	12 (22.2)			20 (38.5)	7 (19.4)		
BSI	35 (28.5)	30 (55.6)			26 (50.0)	22 (61.1)		
Polymicrobial infection = n (%)	29 (23.6)	8 (15.1)	0.287	0.216	11 (21.2)	7 (19.4)	1.000	0.043
Treatment features								
Time to active treatment (median [IQR])	1.00 [0.00, 2.00]	1.00 [0.00, 3.00]	0.013	0.337	1.00 [0.00, 2.00]	1.00 [0.00, 2.50]	0.212	0.326
Treatment duration (median [IQR])	10.00 [7.50, 14.00]	10.00 [7.25, 14.00]	0.692	0.021	10.50 [8.00, 15.50]	10.00 [7.75, 14.00]	0.310	0.083
*Infusion modality (%)*			0.016	0.516			0.040	0.594
Intermittent	26 (21.1)	3 (5.6)			16 (30.8)	3 (8.3)		
Extended	39 (31.7)	26 (48.1)			22 (42.3)	19 (52.8)		
Continuous	58 (47.2)	25 (46.3)			14 (26.9)	14 (38.9)		
Empirical treatment = n (%)	94 (76.4)	48 (88.9)	0.087	0.334	37 (71.2)	31 (86.1)	0.165	0.371
Empirical active treatment = n (%)	87 (70.7)	46 (85.2)	0.063	0.354	31 (59.6)	30 (83.3)	0.033	0.544
*Combination therapy = n (%)*	46 (37.4)	9 (16.7)	0.010	0.480	29 (55.8)	6 (16.7)	0.001	0.891
combination agent being fosfomycin = n (%)	26 (21.1)	3 (5.6)	0.018	0.471	15 (28.8)	3 (8.3)	0.038	0.547
combination agent being aztreonam = n (%)	5 (4.1)	0 (0.0)	0.312	0.291	4 (7.7)	0 (0.0)	0.237	0.408
combination agent being amininoglycoside = n (%)	6 (4.9)	1 (1.9)	0.594	0.168	4 (7.7)	0 (0.0)	0.237	0.408
combination agent being colistin = n (%)	3 (2.4)	3 (5.6)	0.546	0.160	0 (0.0)	2 (5.6)	0.321	0.343
combination agent being tigecycline = n (%)	5 (4.1)	1 (1.9)	0.766	0.131	4 (7.7)	1 (2.8)	0.609	0.222
Laboratory values at infection onset								
Hb (median [IQR])	9.70 [9.00, 11.15]	10.00 [9.00, 12.00]	0.298	0.226	9.60 [8.70, 11.00]	10.00 [8.85, 12.00]	0.254	0.358
WBC x 10^3^ (median [IQR])	11.8 [6.9, 16.3]	13.7 [9.9, 18.9]	0.110	0.098	11.6 [7.5, 15]	13.5 [8.5, 18.9]	0.220	0.357
N% (median [IQR])	87.25 [78.97, 90.60]	86.20 [80.00, 90.00]	0.628	0.042	86.80 [78.97, 90.18]	87.10 [80.00, 89.80]	0.924	0.040
L% (median [IQR])	6.05 [3.00, 13.65]	7.50 [4.70, 11.00]	0.833	0.066	6.10 [4.80, 12.32]	7.20 [3.72, 10.75]	0.821	0.096
PLT x 10^3^ (median [IQR])	172 [127, 265]	190 [131, 295]	0.530	0.144	172.5 [132, 272]	190 [146, 296.5]	0.699	0.120
Creatinine (median [IQR])	0.77 [0.58, 1.21]	0.82 [0.67, 1.35]	0.476	0.298	0.82 [0.59, 1.21]	0.80 [0.70, 1.10]	0.985	0.210
CRP (median [IQR])	14.36 [8.72, 19.79]	56.00 [14.40, 151.00]	< 0.001	1.178	11.40 [6.50, 18.15]	32.70 [12.70, 158.00]	< 0.001	1.109
PCT (median [IQR])	1.75 [0.37, 13.19]	25.65 [5.13, 68.50]	< 0.001	0.587	1.69 [0.54, 12.12]	29.09 [9.00, 67.75]	< 0.001	0.726
GOT (median [IQR])	28.00 [22.00, 39.75]	26.00 [18.50, 48.00]	0.602	0.256	26.00 [20.00, 39.00]	26.00 [19.00, 55.00]	0.620	0.271
GPT (median [IQR])	25.00 [18.00, 45.00]	29.00 [18.50, 47.50]	0.325	0.231	23.00 [15.00, 50.00]	31.00 [18.00, 64.00]	0.163	0.260
Bilirubin (median [IQR])	0.80 [0.42, 1.48]	0.64 [0.56, 1.34]	0.940	0.001	0.70 [0.39, 1.48]	0.67 [0.53, 1.08]	0.723	0.080
INR (median [IQR])	1.16 [1.06, 1.33]	1.21 [1.06, 1.33]	0.666	0.291	1.17 [1.07, 1.29]	1.23 [1.07, 1.37]	0.518	0.381
Fibrinogen (median [IQR])	524.00 [340.50, 623.75]	517.00 [423.00, 565.00]	0.691	0.209	524.00 [351.50, 597.50]	513.00 [447.25, 556.00]	0.951	0.152

BMI: Body mass index;; BSI: bloodstream infection; CKD: chronic kidney disease; COPD: chronic obstructive pulmonary disease; CRP: C reactive protein; CVC: central venous catheter; DM: diabetes mellitus; ECMO: Extracorporeal membrane oxygenation; Hb: hemoglobin; IQR: interquartile range; MV: mechanical ventilation; N: neutrophils; L: lymphocytes; PCT: procalcitonin; PLT: platelets; TPN: total parenteral nutrition; WBC: White blood cells; SMD: standardized mean difference

### Primary outcome– 30-day mortality

**Fig. 2:**
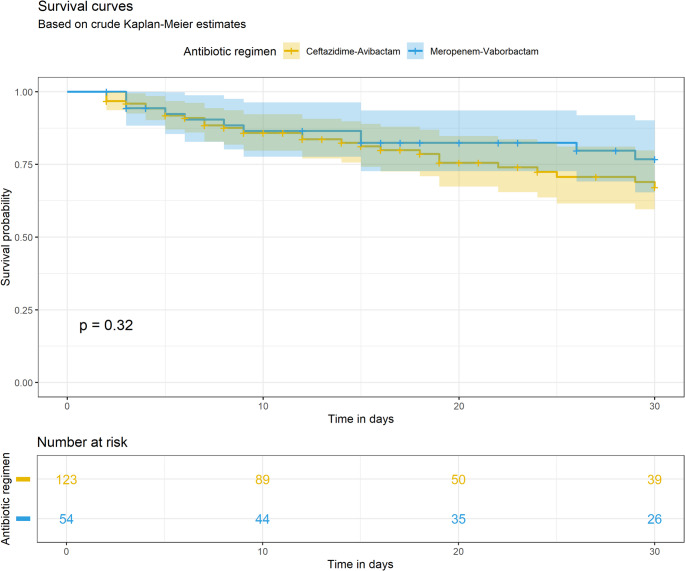
Crude survival curves

In the pre-matching analysis, the crude death rate was 20.4% (11/54) in the M/V group and 24.4% (30/123) in the C/A group. The use of M/V was not associated with a statistically significant impact on survival, HR 0.71 (95% CI 0.35–1.41). Graphically it is depicted in a Kaplan–Meier survival plot: the log-rank test result was *p* = 0.32 (Fig. 2).

In the subgroup analysis, patients with septic shock showed a statistically significant reduction in mortality with M/V (HR 0.47, 95% CI: 0.22–0.99). This result suggests a potential benefit of M/V in this critically ill population but given the limitations of sample size and retrospective design, further studies are needed to confirm this association.

**Fig. 3:**
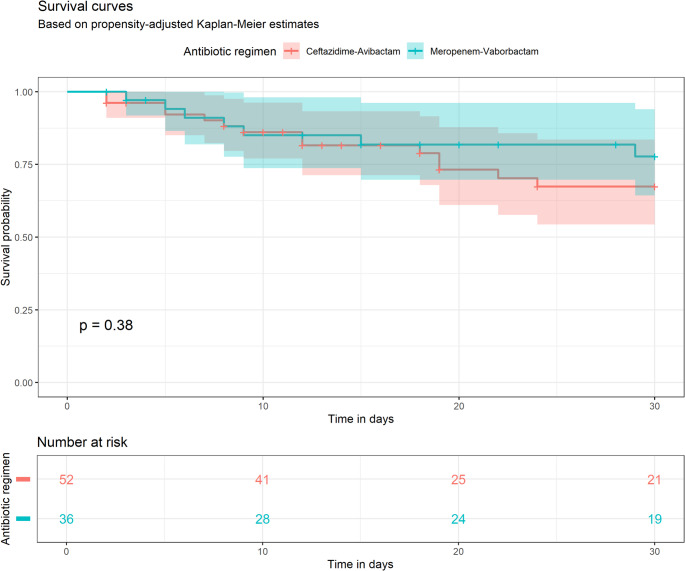
Propensity adjusted survival curves

In the post-matching analysis, the PSM-adjusted 30-day mortality rate among patients treated with M/V was 19.4% (7 out of 36 subjects), opposed to 26.9% (14/52) in patients undergoing targeted treatment with C/A. In the PSM-adjusted Kaplan-Meier analysis, the log-rank test used to gauge the null hypothesis of no difference in survival between the two groups was *p* = 0.38 (Fig. 3). The HR of M/V use on all-cause mortality 0.65 (95% CI 0.55–1.68).

### Secondary outcomes

As for early clinical improvement, in the pre-matching analysis, 66.7% of patients in the M/V group reached the endpoint opposed to 60.2% (74/123) among patients undergoing C/A: the OR for early improvement at 72 h with M/V was 1.32 (95% CI: 0.68–2.59), showing no statistically significant advantage pre-matching. However, after matching, M/V was associated with a statistically significant improvement in early clinical outcomes, with an OR of 2.19 (95% CI: 1.35–3.55), indicating that patients treated with M/V were significantly more likely to show early improvement compared to those treated with C/A (Table 2).

**Table 2:** Odds ratios for early clinical improvement with M/V Pre- and Post-Matching

	OR	95% Confidence Interval
Pre-Matching	1.32	(0.68–2.59)
Post-Matching	2.19	(1.35–3.55)

Post-matching sensitivity analysis for early improvement showed an E-value of 2.32 for the point estimate and 1.6 for the confidence interval, reinforcing the robustness of this finding against unmeasured confounders.

As for secondary infection-related events, descriptive analysis showed non-significant trends in secondary infections, including *Clostridioides difficile* infection, infection relapses, and candidemia, between the two treatments. Rates of secondary infections were similar in both groups, with no statistically significant differences noted.

### Subgroup analysis with regard to 30-day mortality

Seven treatment effect modifiers were tested. In the unmatched cohort, the subgroup of patients with septic shock seemed to benefit from treatment with M/V: HR for mortality was 0.47 (95% CI 0.22–0.99). Across all other levels there were no statistically significant differences between patients undergoing M/V and C/A, although in the majority of instances HRs were below 1, especially in patients undergoing combination therapy (HR 0.24; 95% CI 0.03–1.86) and with concurrent BSI and pneumonia (HR 0.25; 95% CI 0.03–1.88); only exceptions were subjects receiving inappropriate empirical treatment (HR 1.89, 95% CI 0.40–9.20) and continuous infusion therapy (HR 1.04; 95% CI 0.38–2.85) (Table 3).

**Table 3:** Subgroups 30-day mortality (M/V versus C/A) pre-propensity score matching

Subgroup	Pre-matching results	
	HR	95% CI
Under MV	0.73	0.33–1.63
Not under MV	0.56	0.07–4.30
With septic shock	0.47	0.22–0.99
Without septic shock	0.44	0.06–3.35
BSI only	0.88	0.30–2.53
BSI and pneumonia	0.25	0.03–1.88
Pneumonia only	0.82	0.18–3.70
Polymicrobial infection	0.71	0.15–3.31
Monomicrobial infection	0.75	0.34–1.64
Appropriate empirical treatment	0.55	0.26–1.20
Inappropriate empirical treatment	1.89	0.40–9.20
Combination therapy	0.24	0.03–1.86
Monotherapy	0.99	0.44–2.21
Continuous infusion	1.04	0.38–2.85
Extended infusion	0.70	0.24–2.04
Intermittent infusion	0.81	0.09–1.31

BSI: bloodstream infection; MV: mechanical ventilation

As described above, two models were developed for moderation analysis in the context of PSM.

The sample size within each subgroup was limited, contributing to wide confidence intervals.

According to model A, that retained the same population as obtained for the main analysis, in many instances the algorithm did not converge owing to very few subjects and few events (Table 4). In no case was there a statistically significant difference between patients undergoing M/V and C/A, but the former appeared to be more effective in whom receiving appropriate empirical treatment (HR 0.38; 95% 0.10–1.43), whereas in those undergoing monotherapy the opposite was observed (HR 3.30, 95% CI 0.73–14.83) (Table 4).

According to model B based on exact matching ensuing in fair covariate balance (Supplementary materials), that did not reflect the identical population of the main analysis and did not have issues of algorithm convergence, the same pattern described earlier was detected: no statistically significant differences between patients undergoing M/V and C/A across all levels of the variables, and wide confidence intervals due to small subgroup sizes. M/V appeared to be more beneficial in patients with polymicrobial infection (HR 0.17; 95% CI 0.02–1.35), whereas in whom receiving monotherapy a negative impact on mortality was observed (HR 6.15; 95% CI 0.38–98.42) (Table 4).

Of note, both in the unmatched population and in the two models derived from PSM, M/V showed a favorable trend on mortality over M/V compared with C/A in patients with septic shock and under mechanical ventilation. All tests for interaction resulted not significant and were not reported.

**Table 4:** Subgroups 30-day mortality (M/V versus C/A) post-PSM

Subgroup	Model A of moderation analysis				Model B of moderation analysis			
	Sample size		HR	95% CI	Sample size		HR	95% CI
	M/V	C/A			M/V	C/A		
Under MV	36	52	0.44	0.15–1.28	34	48	0.67	0.04–12.11
Not under MV			NC*	NC*			1.50	0.08–27.20
With septic shock	36	52	0.39	0.10–1.56	36	52	0.68	0.03–16.25
Without septic shock			NC*	NC*			1.47	0.06–35.12
BSI only	36	52	0.81	0.19–3.39	34	49	0.76	0.05–12.01
BSI and pneumonia			NC*	NC*			0.42	0.02–8.17
Pneumonia only			NC*	NC*			1.31	0.08–20.53
Polymicrobial infection	36	52	NC*	NC*	34	48	0.17	0.02–1.35
Monomicrobial infection			0.70	0.22–2.26			5.80	0.74–45.16
Appropriate empirical treatment	36	52	0.38	0.10–1.43	33	48	1.21	0.06–24.41
Inappropriate empirical treatment			NC*	NC*			0.82	0.04–16.56
Combination therapy	36	52	NC*	NC*	30	46	0.16	0.01–2.60
Monotherapy			3.30	0.73–14.83			6.15	0.38–98.42
Continuous infusion	36	52	1.00	0.14–7.28	30	46	0.77	0.05–10.75
Extended infusion			2.00	0.20-19.62			0.48	0.05–5.23
Intermittent infusion			NC*	NC*			0.67	0.09–5.32

*Algorithm did not converge (very few cases and few events)

BSI: bloodstream infection; MV: mechanical ventilation

#### Sensitivity analysis

Sensitivity analysis for unmeasured confounding showed an E-value of 1.86 for the point estimate and 1 for the confidence interval in the pre-matching analysis of 30-day survival. After matching, the E-value increased to 2.02 for the point estimate, indicating a moderate robustness of findings to potential unmeasured confounders.

## Discussion

In this retrospective cohort study of 177 critically ill patients with KPC-producing *Klebsiella pneumoniae* invasive infections, we found no significant difference in 30-day all-cause mortality between M/V and C/A. M/V did not show clear mortality benefit, neither pre- nor post-matching. Subgroup analyses of various factors (e.g., septic shock, mechanical ventilation, polymicrobial infection, therapy type, empirical therapy appropriateness, infusion modality, and infection type) revealed no statistically significant mortality differences, although a trend toward lower mortality for M/V in certain subgroups warrants further investigations. Notably, in the matched population, M/V significantly improved early clinical response compared to C/A.

KPC-Kp infections remain a major clinical concern, especially in regions like Southern Europe, Italy, and Greece [29].

Before the introduction of newer β-lactam/β-lactamase inhibitors (BLBLIs) such as C/A and M/V [30], treatment relied on complex regimens often including colistin [3, 31], with mortality rates reaching 40% or higher in vulnerable patients [32]. Carbapenem resistance was associated with increased mortality, largely due to suboptimal, more toxic therapies [33, 34]. However, with the advent of C/A, a recent study found that carbapenem resistance no longer independently predicted mortality [4].

Although initially not approved for carbapenem-resistant infections, C/A rapidly became a benchmark therapy for KPC-Kp, nearly halving mortality compared to older regimens [35, 36].

Other BLBLIs, including M/V and imipenem/cilastatin/relebactam, now broaden the therapeutic landscape [37]. M/V, tested in carbapenem-resistant pathogens in the TANGO II trial, showed improved clinical cure, reduced mortality, and less nephrotoxicity versus the best available therapy [38]. Observational data from Italy further supported M/V’s value, reporting a 31.6% mortality rate and improved outcomes when initiated early [2].

Head-to-head comparisons of these agents are limited. One U.S. retrospective study comparing M/V and C/A found similar success rates, actually slightly higher for M/V (69.2% against 61.9%), but the difference was not significant (*p* = 0.49); notably, resistance to C/A emerged in some patients receiving the drug as monotherapy [10].

Over 65 KPC variants exist, and while M/V may benefit strains resistant to C/A [39–41], resistance to M/V also occurs [6].

Pharmacokinetic differences, such as higher meropenem-vaborbactam penetration in epithelial lining and peritoneal fluids, may influence therapeutic choice [42, 43].

In clinical practice, patient factors like infection severity, comorbidities, and the need for rapid improvement guide therapy selection. Our findings indicate that both M/V and C/A remain viable treatment options in ICU settings. The observed early clinical stabilization with M/V and its association with reduced mortality in septic shock patients should be interpreted cautiously, as these findings require further validation in larger prospective studies. These observations could align with evidence supporting M/V’s use when C/A resistance is suspected or when eraly clinical responses are desired [41, 44–46].

This is the largest known study comparing M/V and C/A for KPC-Kp infections. We applied strict inclusion criteria and used robust statistical methods to ensure a more homogeneous patient population and strengthen the validity of our conclusions.

This study has several limitations due to its retrospective, observational design. Although we used propensity score matching to control for confounders, unmeasured factors may still have influenced the results. The matching predictably reduced the sample size, although creating a more homogeneous population for comparison. The moderation analysis, based on predefined effect modifiers, was exploratory. Smaller subgroup sizes, especially after matching, may have reduced statistical power, potentially explaining the lack of significance in many instances. Moreover, focusing on ICU patients may limit the generalizability of these findings to other patient populations with KPC-producing *K. pneumoniae* infections. Additionally, the presence of polymicrobial infections in a subset of patients could confound the assessment of the specific efficacy of the study drugs against KPC-Kp.

The assessment of “early clinical improvement” is also subject to limitations; as a composite endpoint, it can be difficult to interpret, and the frequency of measuring biomarkers like CRP and procalcitonin may have varied across the participating ICUs, potentially affecting the uniform capture of this outcome.

Eventually, we were not able to formally assess the impact of antibiotic dosing and of minimum inhibitory concentrations, that we will attempt to explore in further studies.

Further research, ideally randomized controlled trials, is needed to confirm these results and evaluate the long-term impacts of M/V versus C/A in critically ill patients. Future studies should consider larger, stratified analyses to better define M/V’s role in high-risk groups, such as those with septic shock or mechanical ventilation. Investigating resistance development and secondary infections may also offer a more comprehensive perspective on optimal CRE treatment strategies in ICUs.

## Conclusion

This study provides comparative observational data on M/V and C/A for the treatment of bloodstream infections and pneumonia caused by KPC-producing *K. pneumoniae* in ICU patients. The results indicate that while no statistically significant differences in 30-day mortality were observed between the two regimens, M/V was associated with a higher rate of early clinical improvement after propensity-score matching. However, this study does not establish a definitive clinical advantage for either agent.

Given the retrospective design and limited sample size, these findings should be interpreted with caution. While the observed association between M/V and improved outcomes in some subgroups, particularly in septic shock patients, is noteworthy, it does not imply causality. Further randomized controlled trials are needed to confirm these results and provide more conclusive evidence regarding the optimal use of M/V and C/A in critically ill patients with KPC-Kp infections.

Ultimately, the choice between M/V and C/A should be guided by patient-specific factors, local resistance patterns, and clinical judgment rather than a presumed superiority of one agent over the other. Future research should focus on larger, prospective studies to better define the roles of these agents in different ICU populations and explore potential combination strategies to optimize patient outcomes.

## Electronic supplementary material

Below is the link to the electronic supplementary material.

Supplementary Material 1

## Data Availability

Data is provided within the manuscript or supplementary information files.
