# Supplementary material for: Head-to-head: meropenem/vaborbactam versus ceftazidime/avibactam in ICUs patients with KPC-producing K. pneumoniae infections– results from a retrospective multicentre study
Source: Infection. 2025 Jul 16;53(6):2645–58. doi: 10.1007/s15010-025-02608-7 (PMC12675711; doi:10.1007/s15010-025-02608-7)
Supplement: Supplementary file 1 — Supplementary Material 1 [file 15010_2025_2608_MOESM1_ESM.pdf]

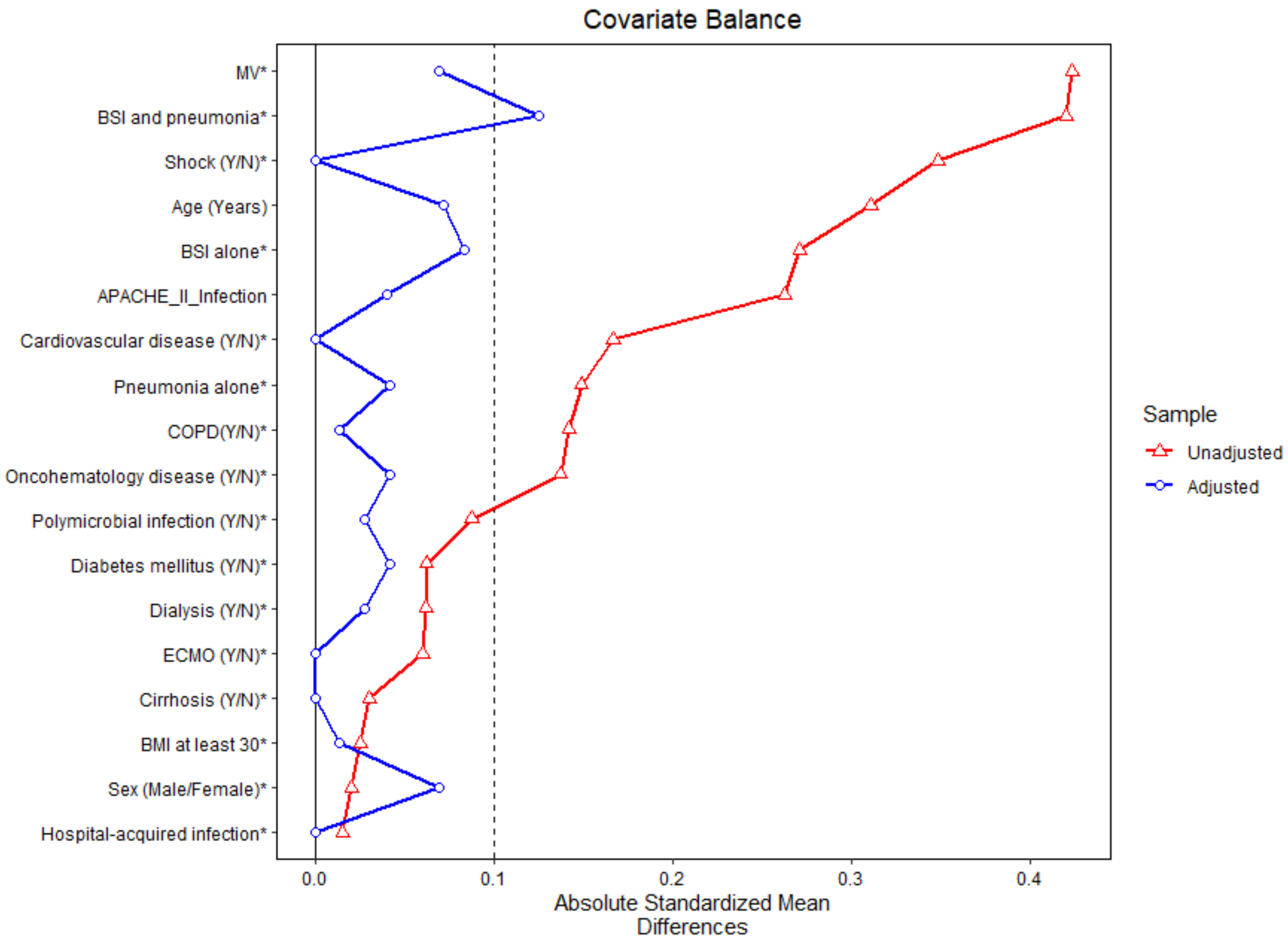
Covariate Balance
MV*
BSI and pneumonia*
Shock (Y/N)*
Age (Years)
BSI alone*
APACHE_II_Infection
Cardiovascular disease (Y/N)*
Pneumonia alone*
COPD(Y/N)*
Oncohematology disease (Y/N)*
Polymicrobial infection (Y/N)*
Diabetes mellitus (Y/N)*
Dialysis (Y/N)*
ECMO (Y/N)*
Cirrhosis (Y/N)*
BMI at least 30*
Sex (Male/Female)*
Hospital-acquired infection*
0.0
0.1
0.2
0.3
0.4
Absolute Standardized Mean Differences
Sample
Unadjusted
Adjusted

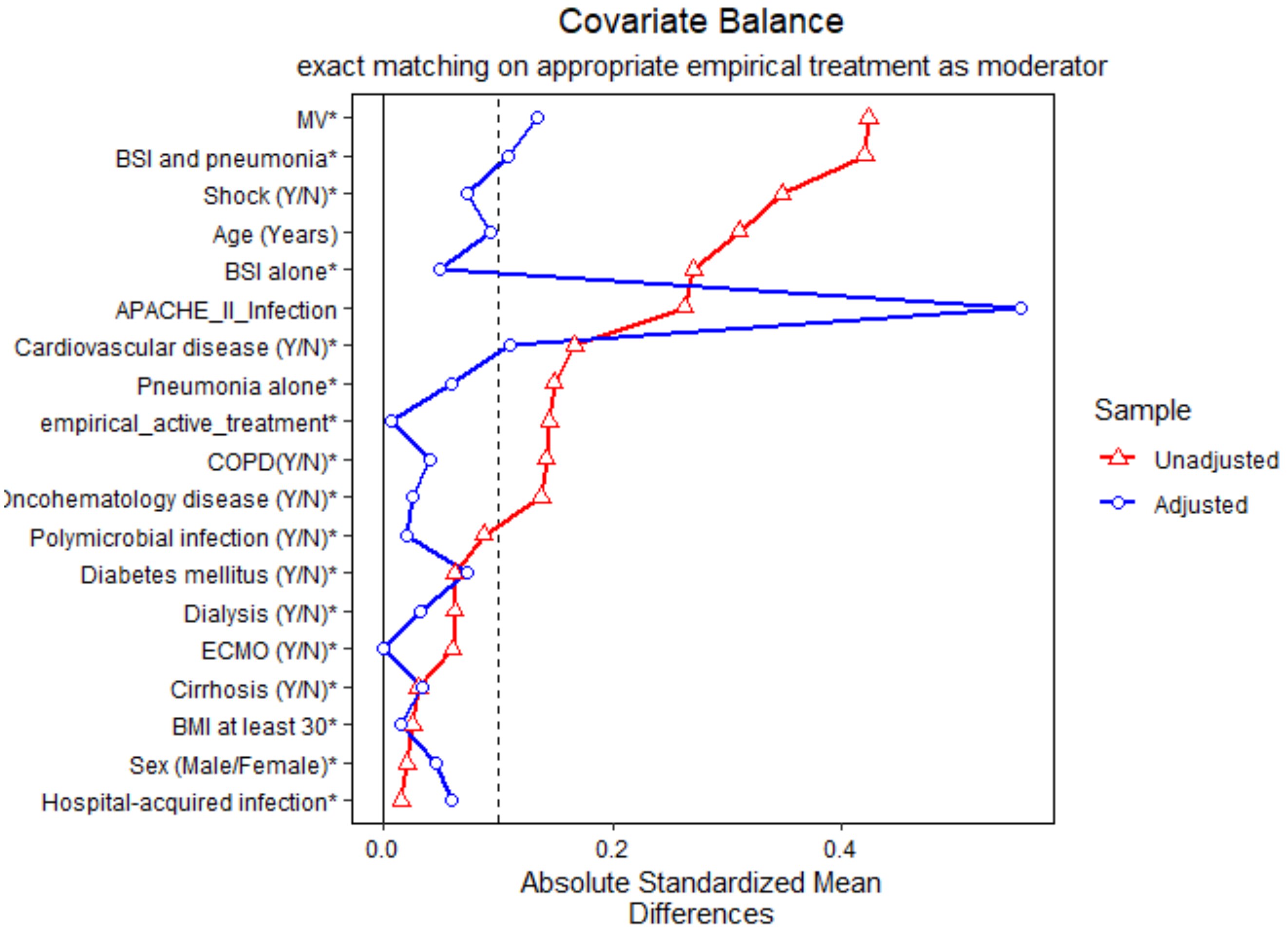

Covariate Balance
exact matching on appropriate empirical treatment as moderator
MV*
BSI and pneumonia*
Shock (Y/N)*
Age (Years)
BSI alone*
APACHE_II_Infection
Cardiovascular disease (Y/N)*
Pneumonia alone*
empirical_active_treatment*
COPD(Y/N)*
)ncohematology disease (Y/N)*
Polymicrobial infection (Y/N)*
Diabetes mellitus (Y/N)*
Dialysis (Y/N)*
ECMO (Y/N)*
Cirrhosis (Y/N)*
BMI at least 30*
Sex (Male/Female)*
Hospital-acquired infection*
0.0
0.2
0.4
Absolute Standardized Mean Differences
Sample
Unadjusted
Adjusted

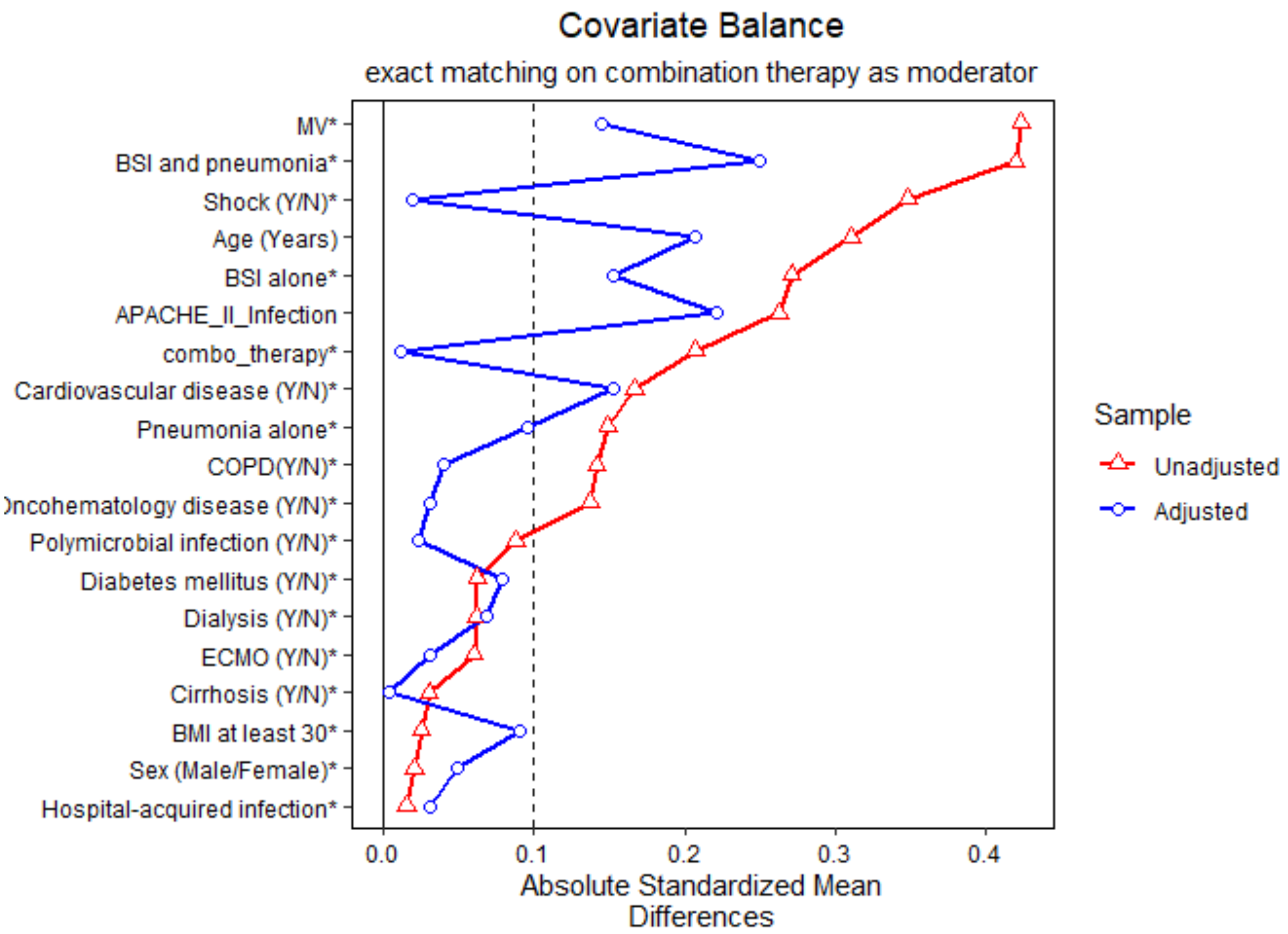
Covariate Balance
exact matching on combination therapy as moderator
MV*
BSI and pneumonia*
Shock (Y/N)*
Age (Years)
BSI alone*
APACHE_II_Infection
combo_therapy*
Cardiovascular disease (Y/N)*
Pneumonia alone*
COPD(Y/N)*
Oncohematology disease (Y/N)*
Polymicrobial infection (Y/N)*
Diabetes mellitus (Y/N)*
Dialysis (Y/N)*
ECMO (Y/N)*
Cirrhosis (Y/N)*
BMI at least 30*
Sex (Male/Female)*
Hospital-acquired infection*
0.0
0.1
0.2
0.3
0.4
Absolute Standardized Mean Differences
Sample
Unadjusted
Adjusted

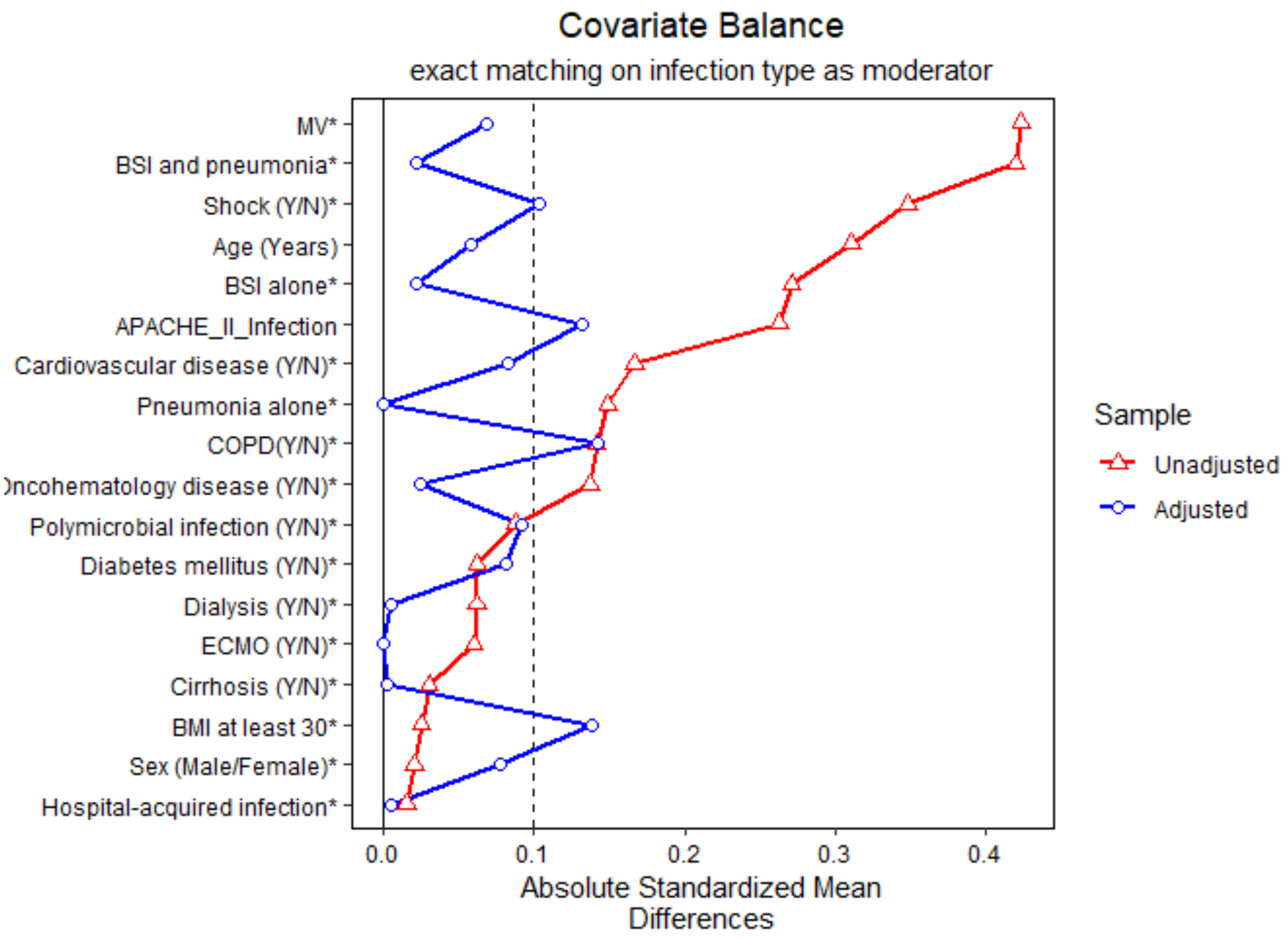

Covariate Balance
exact matching on infection type as moderator
MV*
BSI and pneumonia*
Shock (Y/N)*
Age (Years)
BSI alone*
APACHE_II_Infection
Cardiovascular disease (Y/N)*
Pneumonia alone*
COPD(Y/N)*
Oncohematology disease (Y/N)*
Polymicrobial infection (Y/N)*
Diabetes mellitus (Y/N)*
Dialysis (Y/N)*
ECMO (Y/N)*
Cirrhosis (Y/N)*
BMI at least 30*
Sex (Male/Female)*
Hospital-acquired infection*
0.0
0.1
0.2
0.3
0.4
Absolute Standardized Mean Differences
Sample
Unadjusted
Adjusted

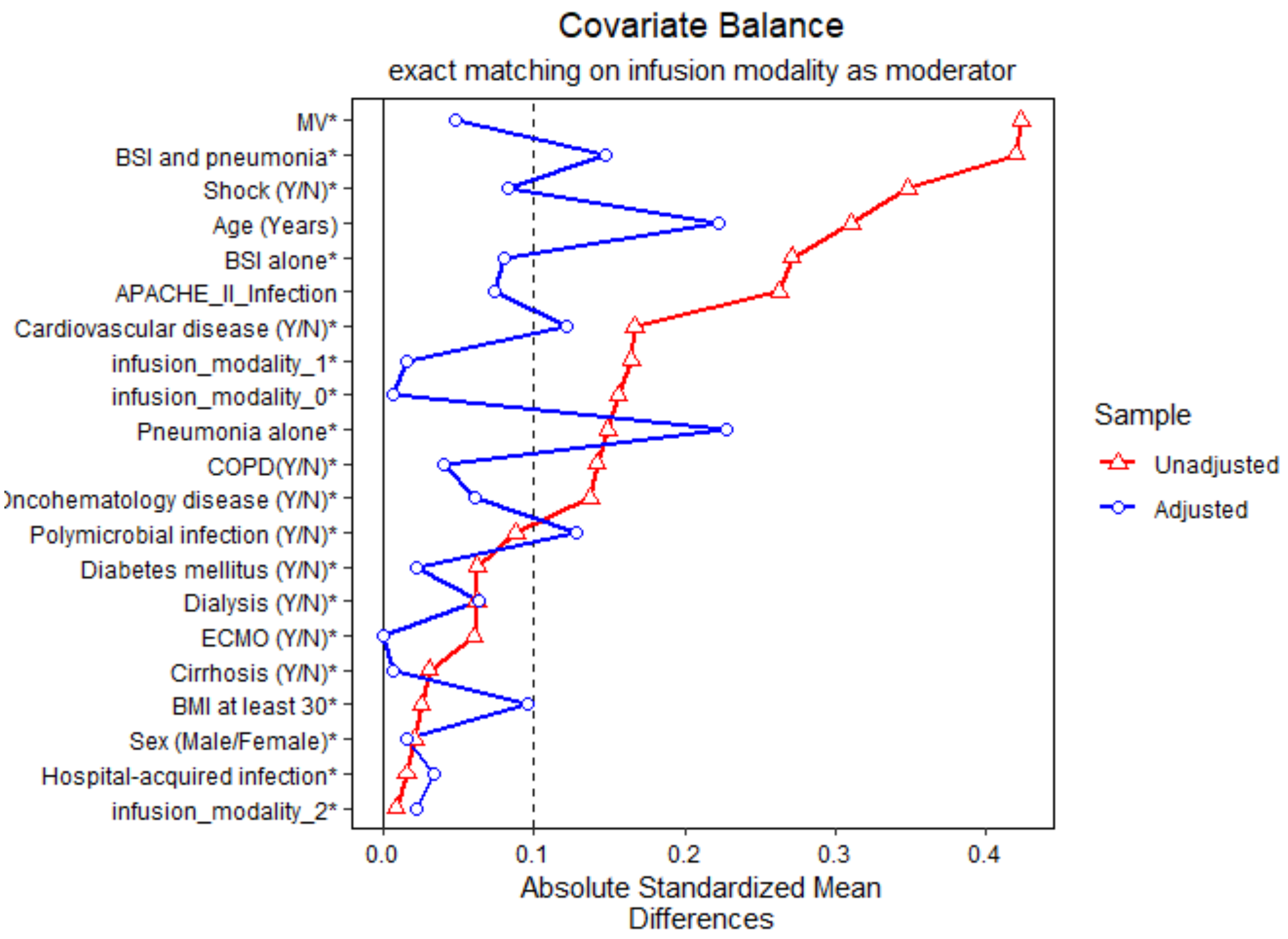
Covariate Balance
exact matching on infusion modality as moderator
MV*
BSI and pneumonia*
Shock (Y/N)*
Age (Years)
BSI alone*
APACHE_II_Infection
Cardiovascular disease (Y/N)*
infusion_modality_1*
infusion_modality_0*
Pneumonia alone*
COPD(Y/N)*
Oncohematology disease (Y/N)*
Polymicrobial infection (Y/N)*
Diabetes mellitus (Y/N)*
Dialysis (Y/N)*
ECMO (Y/N)*
Cirrhosis (Y/N)*
BMI at least 30*
Sex (Male/Female)*
Hospital-acquired infection*
infusion_modality_2*
0.0
0.1
0.2
0.3
0.4
Absolute Standardized Mean Differences
Sample
Unadjusted
Adjusted

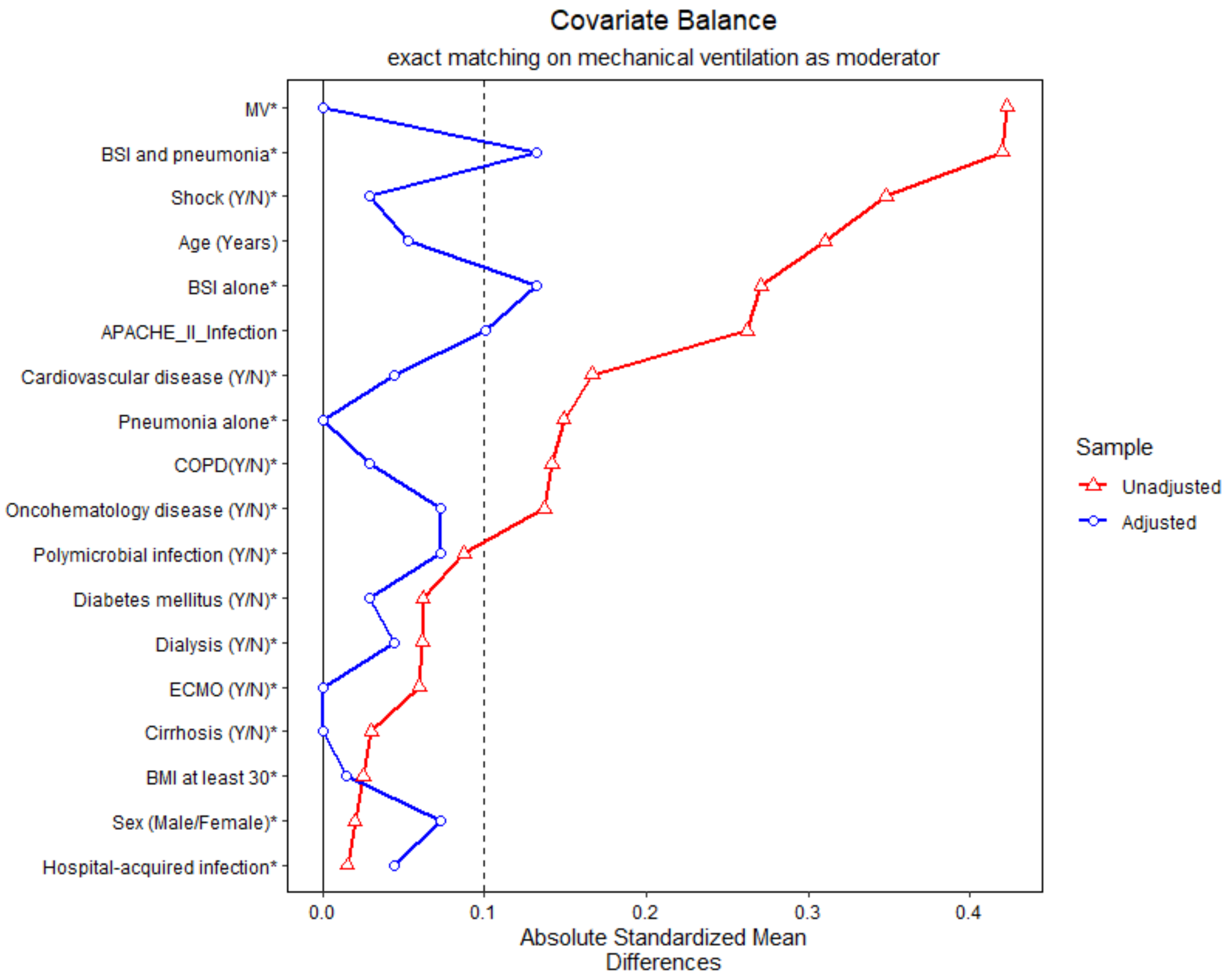

Covariate Balance
exact matching on mechanical ventilation as moderator
MV*
BSI and pneumonia*
Shock (Y/N)*
Age (Years)
BSI alone*
APACHE_II_Infection
Cardiovascular disease (Y/N)*
Pneumonia alone*
COPD(Y/N)*
Oncohematology disease (Y/N)*
Polymicrobial infection (Y/N)*
Diabetes mellitus (Y/N)*
Dialysis (Y/N)*
ECMO (Y/N)*
Cirrhosis (Y/N)*
BMI at least 30*
Sex (Male/Female)*
Hospital-acquired infection*
0.0
0.1
0.2
0.3
0.4
Absolute Standardized Mean Differences
Sample
Unadjusted
Adjusted

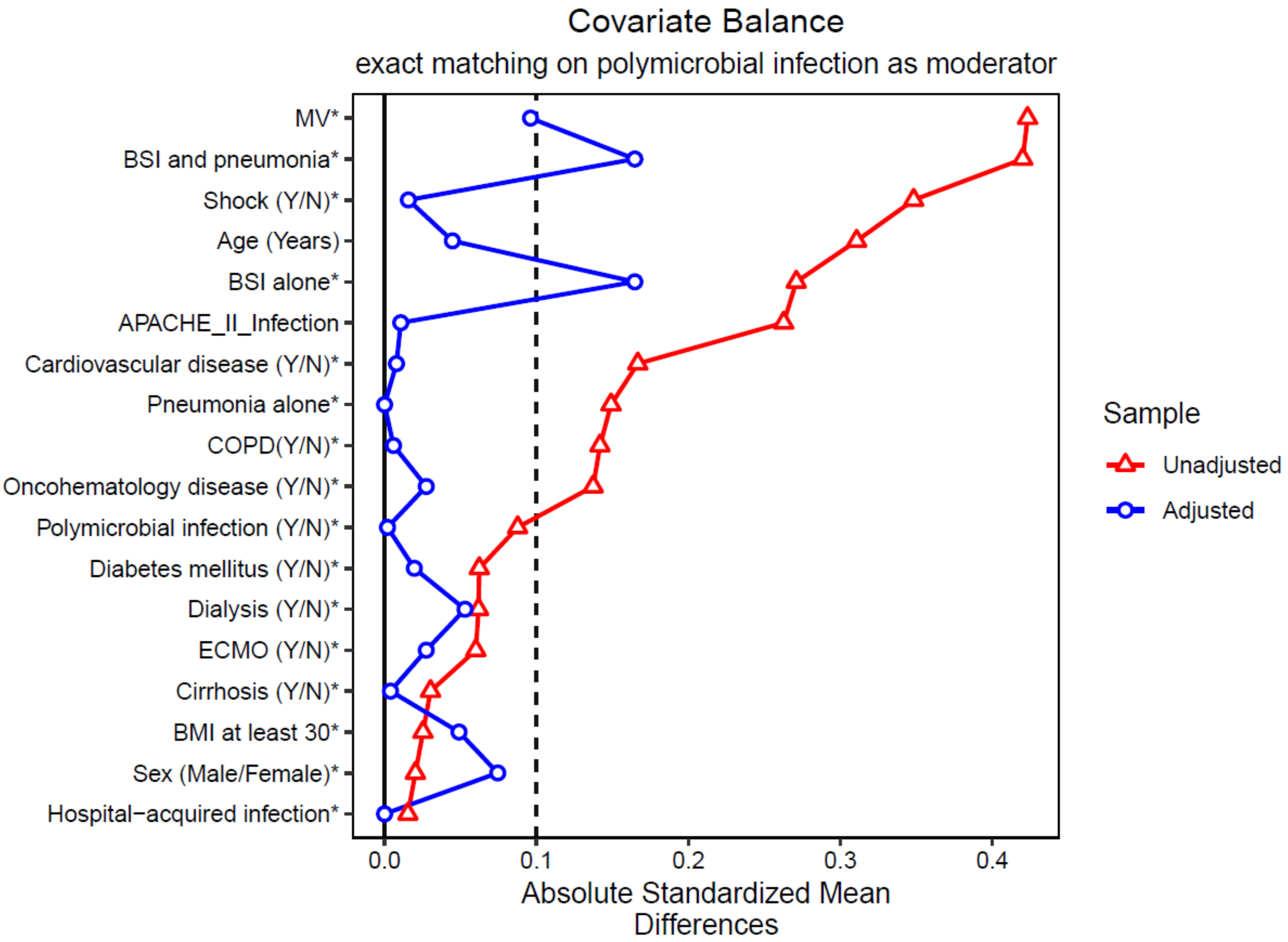
Covariate Balance
exact matching on polymicrobial infection as moderator
MV*
BSI and pneumonia*
Shock (Y/N)*
Age (Years)
BSI alone*
APACHE_II_Infection
Cardiovascular disease (Y/N)*
Pneumonia alone*
COPD(Y/N)*
Oncohematology disease (Y/N)*
Polymicrobial infection (Y/N)*
Diabetes mellitus (Y/N)*
Dialysis (Y/N)*
ECMO (Y/N)*
Cirrhosis (Y/N)*
BMI at least 30*
Sex (Male/Female)*
Hospital−acquired infection*
0.0
0.1
0.2
0.3
0.4
Absolute Standardized Mean Differences
Sample
Unadjusted
Adjusted

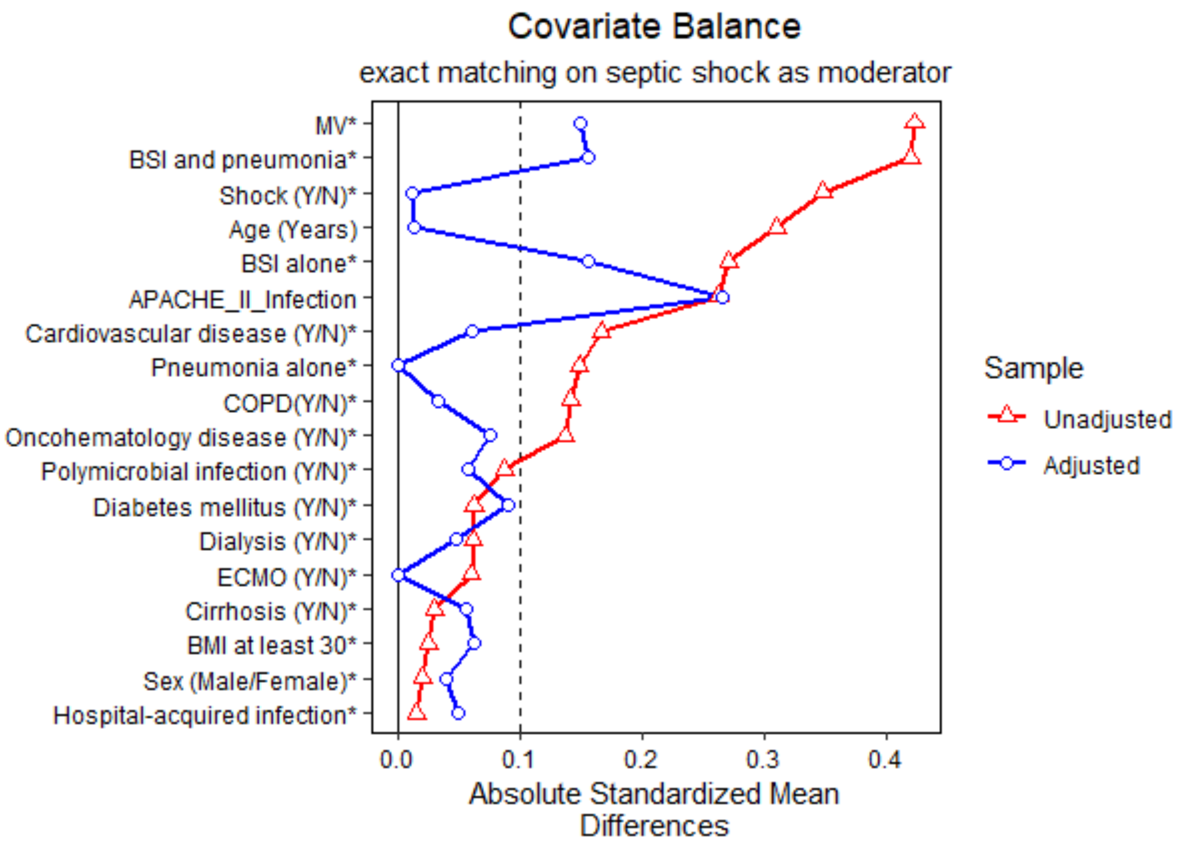

Covariate Balance
exact matching on septic shock as moderator
MV*
BSI and pneumonia*
Shock (Y/N)*
Age (Years)
BSI alone*
APACHE_II_Infection
Cardiovascular disease (Y/N)*
Pneumonia alone*
COPD(Y/N)*
Oncohematology disease (Y/N)*
Polymicrobial infection (Y/N)*
Diabetes mellitus (Y/N)*
Dialysis (Y/N)*
ECMO (Y/N)*
Cirrhosis (Y/N)*
BMI at least 30*
Sex (Male/Female)*
Hospital-acquired infection*
0.0
0.1
0.2
0.3
0.4
Absolute Standardized Mean Differences
Sample
Unadjusted
Adjusted
